# Antimania-Like Effect of *Panax ginseng* Regulating the Glutamatergic Neurotransmission in REM-Sleep Deprivation Rats

**DOI:** 10.1155/2020/3636874

**Published:** 2020-10-17

**Authors:** Kang Hyun Leem, Sang A. Kim, Hae Jeong Park

**Affiliations:** ^1^College of Korean Medicine, Semyung University, Jecheon 27136, Republic of Korea; ^2^Department of Biomedical Science, Graduate School of Kyung Hee University, Seoul 02447, Republic of Korea; ^3^Department of Pharmacology, School of Medicine, Kyung Hee University, Seoul 02447, Republic of Korea

## Abstract

Previous studies have shown the therapeutic properties of ginseng and ginsenosides on hyperactive and impulsive behaviors in several psychiatric diseases. Herein, we investigated the effect of *Panax ginseng* Meyer (PG) on hyperactive/impulsive behaviors in a manic-like animal model, sleep deprivation (SD) rats. Male rats were sleep-deprived for 48 h, and PG (200 mg/kg) was administered for 4 days, from 2 days prior to the start of SD to the end date of SD. The elevated plus maze (EPM) test showed that PG alleviated the increased frequency of entries into and spent time within open arms by SD. In order to investigate the molecular mechanism on this effect of PG, we assessed differentially expressed genes (DEGs) in the prefrontal cortex of PG-treated SD rats using RNA sequencing (RNA-seq) and performed gene-enrichment analysis for DEGs. The gene-enrichment analysis showed that PG most prominently affected the glutamatergic synapse pathway. Among the glutamatergic synapse pathway genes, particularly, PG enhanced the expressions of glutamate transporter Slc1a3 and Slc1a2 reduced in SD rats. Moreover, we found that PG could inhibit the SD-induced phosphorylation of the NR2A subunit of the NMDA receptor. These results suggested that PG might have a therapeutic effect against the manic-like behaviors, regulating the glutamatergic neurotransmission.

## 1. Introduction

Sleep disturbance is the most frequent symptom in manic episodes and a relapse factor of manic episodes in bipolar disorder (BPD) [1, 2]. In rodents, sleep deprivation (SD) has been considered as an environmental mania model [3, 4]. Indeed, in rats and mice, SD rodents have shown manic-like behaviors, including locomotor hyperactivity, hypersexuality, irritability, and aggressiveness, as observed during the manic phase in patients with BPD [3, 4]. In addition, treatments of mood stabilizers such as lithium and valproic acid, which are have been used for relieving in the manic episodes in BPD, reduced these manic-like behaviors in SD animals [5–7]. Neurochemical alterations in SD animals were also represented, similar to patients during the manic phase in BPD. In the brains of SD animals, the dopaminergic hyperactivity, the increased activity of protein kinase C, and the decreased levels of brain-derived neurotrophic factor (BDNF) were observed [5, 8–10].

Ginseng is a popular herbal medicine widely used for multiple pharmacological functions such as anticancer, antioxidant, antistress, and antiaging effects [11]. Previous studies have also reported that ginseng has antidepressant and antipsychotic effects. Ginseng extract and its ingredient ginsenosides increased the level of BDNF and its signaling pathway molecules and activated the neurogenesis in the hippocampus of depressive animals exposed to chronic mild stress [12, 13]. In addition, ginseng decreased the schizophrenic symptoms in prenatally stressed neurodevelopmental and ketamine-treated schizophrenic rodents, regulating the neurodevelopmental genes and the levels of dopamine and 5-hydroxytryptamine [14, 15].

Based on various pharmacological properties of ginseng including beneficial effects on psychiatric diseases, in this study, we examined the effect of *Panax ginseng* Meyer (PG) extract on manic-like behavior in SD rats. In addition, in order to identify the molecular mechanism on the effect of PG on manic behaviors, we assessed differentially expressed genes (DEGs) by PG treatment in the prefrontal cortex of SD rats using RNA sequencing (RNA-seq). Among the DEGs, we particularly paid attention to the genes belonging to the glutamatergic synapse pathway, which showed the lowest *p* value in the gene-enrichment analysis on DEGs based on the Kyoto Encyclopedia of Genes and Genomes (KEGG) pathways. We also focused on amphetamine addiction pathway genes because the amphetamine treatment in rodents has been widely used as a pharmacological mania model. The effect of PG on mania and BPD was further studied and discussed, focusing on the genes belonging to the glutamatergic synapse and amphetamine addiction pathways.

## 2. Materials and Methods

### 2.1. Preparation of PG

PG was purchased from Cheongsongyinsamsa (Kumsan, Chungnam, Korea) and was ground to powder. It (300 g) was extracted twice with 3 L of 70% ethyl alcohol for 1 day in a room temperature, filtered through filter paper (Advantec, Bunkyo-Ku, Tokyo, Japan), concentrated by rotary evaporator (N-1200BS, Eyela, Tokyo, Japan), and then freeze-dried (Freezone 6, Labconco, Kansas, MO, USA). The yield of freeze-dried PG was calculated to be 16.7%.

### 2.2. Animals

Male Sprague-Dawley rats (150-200 g; Central Lab. Animal Inc., Seoul, Republic of Korea) were maintained under a 12-h light/dark cycle (light on at 7:00 AM and off at 7:00 PM) at a standard temperature (25 ± 2°C) with food and water freely available. All animal experiments were conducted in accordance with the animal care guidelines of the National Institute for Health (NIH) Guide and approved by the Animal Care and Use Committee at Semyum University (smecae17-07-02).

### 2.3. SD and PG Treatment

For REM-SD, the modified multiple platform method was used [10, 16]. We randomly divided rats into control (non-SD), SD, and PG-treated SD groups. The rats of the SD and PG-treated SD groups were put in a chamber with six small platforms (6 cm diameter) surrounded by water (*n* = 4 per chamber). When the rats enter REM sleep, the rats were awakened when they touch the water by diminished muscle tone. The rats of the control group were put in a chamber with six large platforms (15 cm diameter) (*n* = 4 per chamber). On large platforms, the rats could sleep on. But those are also surrounded by water and not enough for rats to walk around on. The water was filled up to about 2 cm below the surfaces of the platforms, and the rats could move across platforms within the chamber with large or small platforms. Food and water were available ad libitum. All rats remained in the chambers for 48 h.

PG has been used as a treatment in CNS disease rats such as psychiatric and neurological diseases at doses of 100-200 mg/kg [17–19]. Based on these previous reports, we treated PG (200 mg/kg, in a concentration of 40 mg/ml in distilled water, p.o.) to rats of the PG-treated SD group, 5 times for a total period of 4 days, from 2 days prior to the start of SD (9:00 AM) to the end of SD at 9:00 AM. To the rats of the control and SD groups, distilled water was taken in the same manner. After 48 h, the rats of all groups were sacrificed. The prefrontal cortex from each rat was dissected out, weighed, and kept frozen until analysis.

### 2.4. Elevated Plus Maze (EPM) Test

After SD for 48 h, the EPM test was carried out in the manner of the previous study [20]. The EPM test apparatus was consisted of two open arms (30 × 10 cm) and two enclosed arms (30 × 10 × 30 cm, with an open roof) of black wood and raised to a height of 50 cm. After habituation in the testing room for 20 min, the rats were put in the center of a cross-maze facing the open arms. For 5 min (test session), we checked the time spent and the numbers of entries into the open or closed arms.

### 2.5. RNA Isolation and RNA-Seq

The next-generation sequencing was performed using the RNA pooled from 3 rats per group. The total RNA was isolated from the prefrontal cortex samples pooled in each group using TRIzol. RNA integrity was measured using an Agilent 2100 Bioanalyzer (Agilent Technologies, Inc., Santa Clara, CA, USA).

The cDNA libraries were constructed using the TruSeq RNA Library kit on 1 *μ*g of total RNA according to the following process: extraction of polyA-selected RNA, fragmentation of RNA, reverse transcription using random hexamer, and 100 nt paired-end sequencing using Illumina HiSeq4000. We quantified the libraries through qPCR using an Agilent Technologies 2100 Bioanalyzer.

The low quality and adapter sequences were excluded from the raw reads of the sequencer. And then, the reads were aligned to the *Rattus norvegicus* (UCSC Baylor 3.4/rn4) using HISAT v2.0.5 [21]. The reference genome sequence of *Rattus norvegicus* and annotation data were acquired from the NCBI. The aligned reads were assembled into the transcripts, and their abundance was estimated using StringTie v1.3.3d [22, 23]. The relative abundance was estimated as fragments per kilobase of exon per million fragments mapped reads (FPKM) of transcript and gene expressed in each group. The RNAseq data has been uploaded to the public repository Sequence Read Archive (SRA) (https://submit.ncbi.nlm.nih.gov; submission ID: SUB8063770).

### 2.6. Statistical Analysis of Gene Expression Level

In order to select differentially expressed genes, the relative abundances for each gene were compared between groups. Genes whose FRKM was 0 in any of the groups were excluded from the analysis. After 1 was added to the FPKM values of genes, the values were converted based on log_2_ and then were subjected to quantile normalization. We determined the differential expression data by ∣fold change (FC) | ≥2 and independent *t*-test. In order to measure the similarity, hierarchical clustering for the DEG set was conducted using complete linkage and Euclidean distance. The gene-enrichment analysis for DEGs was also conducted based on the KEGG pathway (https://www.genome.jp/kegg/) database. Enrichment *p* values on KEGG pathway terms were calculated based on a modified Fisher's exact test. And then, the false discovery rate (FDR-) adjusted *p* values using the Benjamini-Hochberg algorithm were calculated. All data analysis on RNA-seq was conducted using R 3.4.3 (http://www.r-project.org/).

### 2.7. Quantitative Real-Time PCR (qRT-PCR)

cDNA was synthesized from total RNA using a 1st strand cDNA Synthesis Kit (BioAssay Co., Daejeon, Republic of Korea) according to the manufacturer's instructions. qRT-PCR was conducted using Real-Time PCR EvaGreen Kit (SolGent, Daejeon, Republic of Korea) and specific primers on each gene (Table S3). The analyses were performed using a StepOnePlus Real-Time PCR System (Applied Biosystems Inc., Carlsbad, CA, USA). The relative quantification of mRNA transcripts was calculated based on the 2^−*ΔΔ*CT^ method [24]. The expressions of UBC as a housekeeping gene were used for the analysis of relative expression levels of other genes [25].

### 2.8. Immunoblot

The prefrontal cortex dissected from each rat was homogenized in RIPA buffer with 1× protease/phosphatase inhibitor cocktail (Cell Signaling Technology, Beverly, MA, USA). For assessing the protein concentration, the Bradford reagent (Sigma-Aldrich, St. Louis, MO, USA) was used. After separated on sodium dodecyl sulfate (SDS-) polyacrylamide gels, proteins of 50 *μ*g were transferred onto a nitrocellulose membrane (Amersham Biosciences, Uppsala, Sweden). The membranes were incubated with mouse solute carrier family 1 (glial high-affinity glutamate transporter), member 2 (Slc1a2; also known as Eaat2) (Merck Millipore Darmstadt, Germany), rabbit Slc1a3 (also known as Eaat1), rabbit activity-regulated cytoskeleton-associated protein (Arc), rabbit *N*-methyl-D-aspartate (NMDA) receptor subunit 2A (NR2A), rabbit phospho-NR2A, rabbit NR2B (Y1325, Abcam, Cambridge, UK), and rabbit phospho-NR2B (Y1472; Cell Signaling Technology). Then, the membranes were further incubated with horseradish peroxidase-conjugated antimouse or antirabbit IgG (Santa Cruz Biotechnology, Santa Cruz, CA, USA). ECL substrate (Bio-Rad Laboratories, Hercules, CA, USA) was used for the visualization of protein bands. The band intensity was quantified using the ImageJ image analysis software (version 1.4; NIH).

### 2.9. Statistical Analysis

The results are expressed as mean ± standard error of the mean (SE). The data were analyzed by one-way ANOVA followed by the LSD post hoc test, using the IBM SPSS Statistics 23 (SPSS Inc., Chicago, IL, USA). Values of *p* < 0.05 were considered as statistically significant.

## 3. Results

### 3.1. Effect of PG on Manic-Like Behavior of SD Rats

**Figure 1:**
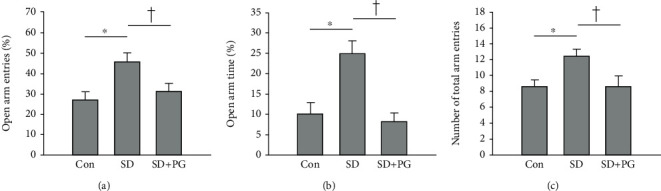
Effects of *Panax ginseng* (PG) on manic-like behaviors in sleep deprivation (SD) rats. Manic-like behaviors in sleep deprivation rats were measured using the elevated plus-maze (EPM) test. Impulsive behavior was indicated by (a) the percentage of open arm entries and (b) percentage of time spent in the open arms. (c) Hyperactive locomotion was indicated by the number of total entries into the open and closed arms. The results are presented as the mean ± SE of two independent experiments (*n* = 4 for each group per each experiment). ^∗^*p* < 0.05 compared to the control group; ^†^*p* < 0.05 compared to the SD group. PG, PG.

The effect of PG on the hyperactive- and impulsive-like behaviors was assessed in SD rats using the EPM test. In the EPM test, the increased frequencies of open arm entries and the spent time in open arms have been considered as a sign of impulsive-like behavior [26, 27]. As shown in Figure 1, the SD rats entered the open arms more frequently and spent more time in the open arms than the control rats. In comparison, the PG-treated SD rats showed the decreased frequency of entries into and spent time within the open arms compared to the SD rats (Figures 1(a) and 1(b)). This result indicated that the PG treatment alleviated the increased impulsivity in SD rats. In addition, the PG treatment reduced the hyperlocomotion shown in SD rats. The frequency of the total entries to the open and close was increased in SD rats compared to control rats, whereas PG-treated SD rats revealed a lower frequency of total entries than SD rats (Figure 1(c)).

### 3.2. Altered Gene Expression by PG in the Prefrontal Cortex of SD Rats

In order to identify the alteration of gene expressions by PG treatment in SD rats, we performed RNA-seq in the prefrontal cortex of the control, SD, and PG-treated SD rats. Through RNA-seq, we acquired the expression data on 13,254 genes. In the DEG analysis on RNA-seq result, we selected genes showing ∣FC∣ of 2.0 or greater and the a *p* value of 0.05 or less in comparisons among groups. A total of 66 DEGs were identified in the control vs. SD comparison (Table S1). The expressions of 31 genes were upregulated in the SD rats compared to control rats, and the expressions of 35 genes were downregulated. In the SD vs. PG-treated SD comparison, 94 DEGs were identified (Table S2). PG treatment increased the expressions of 56 genes and decreased the expressions of 38 genes in SD rats. In order to determine the effect of PG on SD, we focused on 94 DEGs identified in the SD vs. PG-treated SD comparison and carried out a gene-enrichment analysis on 94 DEGs. Through the enrichment analysis based on the KEGG pathway database, 26 significant pathways were detected (FDR-corrected *p* < 0.05; Table 1). Among the pathways, the glutamatergic synapse pathway showed the most significant enrichment. The glutamatergic synapse pathway genes altered by PG treatment in SD rats included Slc1a2, Slc1a3, adenylate cyclase 1 (Adcy1), glutamate ionotropic receptor kainate type subunit 3 (Grik3), glutamate ionotropic receptor NMDA type subunit 3A (Grin3a), and G protein subunit gamma 2 (Gng2). In PG-treated SD rats, the expressions of all of these genes were elevated, compared to SD rats (Table 2). We also paid attention to the amphetamine addiction pathway (Table 1), because the amphetamine treatment in rodents has been widely used as a pharmacological mania model. Among the genes belonging to the amphetamine addiction pathway, the expressions of calcium/calmodulin-dependent protein kinase II alpha (Camk2a) and Grin3a were increased in PG-treated rats compared to SD rats, whereas the expression of Arc was decreased (Table 2). Among the genes belonging to the glutamatergic synapse or amphetamine addiction pathways, Slc1a3, Gng2, and Arc were also identified as DEGs in the control vs. SD comparison. In the SD rats, the expression of Slc1a3 and Gng2 was downregulated, and the expression of Arc was upregulated, compared to control rats (Table 2).

### 3.3. Effect of PG on the Expressions of the Glutamatergic Synapse and Amphetamine Addiction Pathway Genes in SD Rats

**Figure 2:**
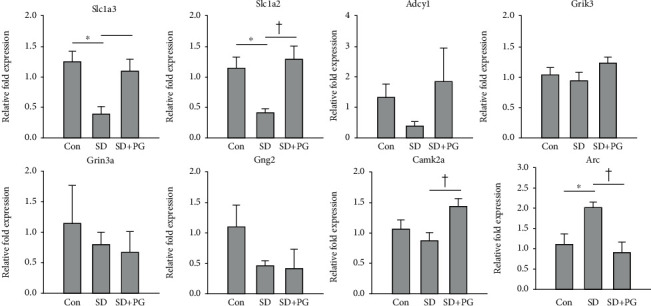
Quantitative real-time PCR (qRT-PCR) validation of the glutamatergic synapse and amphetamine addiction pathway genes. Expressions of the glutamatergic synapse and amphetamine addiction pathway genes were determined using the qRT-PCR in the prefrontal cortex of the control, sleep deprivation (SD), and *Panax ginseng* (PG)-treated SD rats. The histogram reveals the expression levels of genes as mean ± SE. Expression levels were normalized against ubiquitin C (Ubc). The experiments were repeated in duplicates (*n* = 4 for each group per experiment). ^∗^*p* < 0.05 compared to the control group; ^†^*p* < 0.05 compared to the SD group.

To validate the RNA-seq result, we examined the expressions of 8 genes belonging to the glutamatergic synapse and amphetamine addiction pathways in the prefrontal cortex of the control, SD, and PG-treated SD rats using qRT-PCR. As shown in Figure 2, the mRNA expressions of Slc1a3 and Slc1a2 were decreased in SD rats compared to the control rats, and the PG treatment alleviated the decreases. In contrast, SD elevated the expression of Arc, and the PG treatment inhibited the elevation of Arc in SD rats. The expression level of Camk2a was higher in the PG-treated SD rats than in SD rats, but the difference of the expressions between the control and SD rats was not observed. Although our RNA-seq result showed that the expressions of Adcy1, Grik3, Grin3a, and Gng2 were upregulated in PG-treated SD rats compared to SD rats, the significant difference of their expressions was not detected in the qRT-PCR analysis.

### 3.4. Effect of PG on the Protein Expressions of Slc1a2, Slc1a3, and Arc in SD Rats

**Figure 3:**
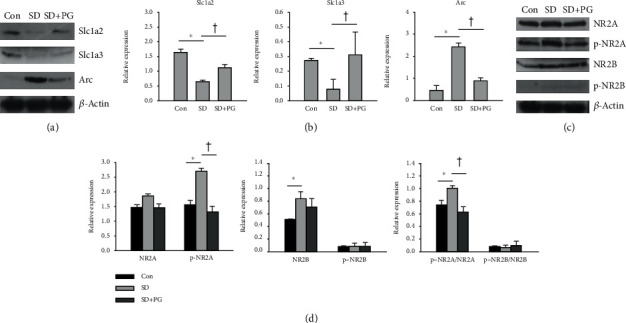
Effect of *Panax ginseng* (PG) on the expression of Slc1a2, Slc1A3, and Arc and the phosphorylation of the NMDA receptor in sleep deprivation (SD) rats. (a) The expressions of Slc1a2, Slc1A3, and Arc and (b) the phosphorylations of the NR2 subunits of the NMDA receptor were determined using immunoblotting in the prefrontal cortex of the control, SD, and PG-treated SD rats. The left and middle histograms represent the protein expression and the phosphorylation levels divided by the expression level of *β*-actin as mean ± SE. The right histogram shows the ratio of the phosphorylation levels to the protein expression levels. *β*-Actin expression was used as an internal control. The experiments were repeated in duplicates (*n* = 3 for each group per experiment). ^∗^*p* < 0.05 compared to the control group; ^†^*p* < 0.05 compared to the SD group.

We also examined the protein expressions of Slc1a2, Slc1a3, and Arc in the prefrontal cortex of the control, SD, and PG-treated SD rats (Figures 3(a) and 3(b)). The protein expressions of Slc1a2 and Slc1a3 were reduced in SD rats, whereas the PG treatment restored the reduction in SD rats. In contrast, SD enhanced the protein expression of Arc, whereas the PG treatment in SD rats suppressed the enhanced expression of Arc.

### 3.5. Effect of PG on the Activation of NMDA Receptors in SD Rats

In order to determine the effect of PG on the activation of NMDA receptor, which is a key receptor in neurotransmission through the glutamatergic synapse and amphetamine addiction pathways, we assessed the expression and phosphorylation levels of NR2A and NR2B subunits of the NMDA receptor in the prefrontal cortex of the control, SD, and PG-treated SD rats. As shown in Figures 3(c) and 3(d), SD and PG treatment did not affect the protein expression of NR2A. However, SD could elevate the phosphorylation of NR2A, whereas PG inhibited the SD-induced phosphorylation of NR2A. In addition, SD increased the protein expression of NR2B. However, the PG treatment did not suppress the expression of NR2B. Neither SD nor PG treatment affects the phosphorylation of NR2B. These results indicate that the PG treatment could inhibit the SD-induced activation of the NR2A subunit of the NMDA receptor in the prefrontal cortex, but not the NR2B subunit.

## 4. Discussion

In this study, we found that PG could attenuate the manic-like behaviors shown in SD rats. In order to identify the mechanism of the effect of PG in SD rats, we assessed the alteration of molecules by PG treatment in the prefrontal cortex of SD rats. PG prominently altered the expressions of the glutamatergic synapse and amphetamine addiction pathway genes in SD rats. In particular, PG alleviated the decreased expressions of Slc1a2 and Slc1a3 and the increased expression of Arc shown in SD rats. These alleviations by PG in SD rats were also observed in the protein levels. Moreover, PG could inhibit the SD-induced phosphorylation of the NR2A subunit of the NMDA receptor.

BPD patients in a manic state represent the characteristic behaviors such as hyperactivity, impulsivity, and aggressiveness [28, 29]. Rodent models of mania have also shown the hyperactive- and the impulsive-like behaviors, increasing the total number of entries into the arms and the frequencies of the open arm entries/the spent time in the open arms in the EPM test [30–32]. Also, in our SD rats, the hyperactive and impulsive mania-like behaviors were observed through the EPM test, similar to the mania rodent models of previous studies. Interestingly, PG treatment significantly reduced these mania-like behaviors. A lot of studies have reported the effect of ginseng and ginsenosides on the hyperactive/impulsive behaviors in psychiatric diseases and conditions. Clinical studies showed that PG and Korean red ginseng (KRG) alleviated the hyperactivity/impulsivity in patients with attention deficit hyperactivity disorder (ADHD) [33, 34]. Additionally, in animal studies, ginsenosides relieved the hyperactive behaviors, along with reducing the behavioral sensitization and conditioned place preference in cocaine- [35], methamphetamine- [36], morphine- [37], and nicotine-treated mice [38, 39]. In addition to these behavioral effects, several studies reported that ginseng and ginsenosides could suppress the enhanced central dopaminergic transmission including dopamine-releasing and/or binding to dopamine receptors [36–39]. However, the mechanism on the alleviating effect of ginseng or ginsenosides against manic behaviors is still not largely elucidated.

In our study, through RNA-seq, we found that PG could regulate potently the glutamatergic synapse pathway genes in the prefrontal cortex of SD rats. The expressions of Slc1a3, Slc1a2, Adcy1, Grik3, Grin3a, and Gng2 were increased by the PG treatment in SD rats. We also paid attention to the amphetamine addiction pathway genes such as Camk2a, Arc, and Grin3a. The expressions of Camk2a and Grin3a were upregulated by the PG treatment in SD rats, whereas the expression of Arc was downregulated. Among these genes, we expected that Slc1a3, Gng2, and Arc might be candidate genes related to the alleviating effect of PG on manic-like behaviors of SD rats. Intriguingly, these genes were not only detected as DEGs in the comparison between SD and control but also PG treatment restored the expression changes of these genes in SD rats. In qRT-PCR, we also observed the expression changes on Slc1a3 and Arc, similar to the RNA-seq result, but not on Gng2. SD attenuated the expression of Slc1a3 and enhanced the expression of Arc, whereas PG relieved SD-induced expression changes. Moreover, we found the additional candidate gene Slc1a2. Although in the DEG analysis between the control and SD, Slc1a2 was excluded by ∣FC∣ cutoff of ≥2 (FC = −1.7; Table 2); qRT-PCR showed that the expression of Slc1a2 was significantly reduced by SD and PG ameliorated the reduced expression, consistent with our RNA-seq result. However, differently from the RNA-seq result, the expressions of Adcy1, Grik3, Grin3a, and Gng2 were not upregulated by PG treatment in SD rats. This inconsistency may be attributed to the expression volume of the gene that was the geometric mean of the two groups' expression levels. Even though ∣FC∣ might be different by twofold, the expression change of gene with higher volume may be more credible [40]. In our RNA-seq result, Slc1a3, Slc1a2, Camk2a, and Arc showed higher expression volumes than Adcy1, Grik3, Ginr3a, and Gng2 (data not shown). Although the expression changes of Adcy1, Grik3, Grin3a, and Gng2 were not detected in qRT-PCR (Figure 2); instead, we could acquire the confidence on the expression changes of Slc1a3, Slc1a2, Camk2a, and Arc. We focused on Slc1a3, Slc1a2, and Arc and examined their protein expressions. SD reduced the protein expressions of Slc1a3 and Slc1a2 and elevated the expression of Arc. In comparison, PG treatment inhibited SD-induced these expression changes (Figures 3(a) and 3(b)).

The concentrations of glutamate in the extracellular space are maintained by glutamate transporters. Glutamate transporters, Slc1a and Slc1a3, were potently located in the plasma membranes of astrocytes and oligodendrocytes. Those remove approximately 95% of the extracellular glutamate [41]. The glutamatergic neurotransmission has been involved in the pathophysiology and treatment of mania and BPD. The elevated levels of glutamate have been reported in patients with BPD [42, 43]. A mood stabilizer, valproic acid, was reported to increase the levels of Slc1a2 and Slc1a3 and the capacity of glutamate uptake in the rat hippocampus [44] and also to decrease the glutamate levels in mice whole brain [45]. Given these reports, PG may attenuate the synaptic glutamate level, enhancing the glutamate uptake via increasing the expression levels of Slc1a and Slc1a3 in SD rats. Thus, it may contribute to suppressing the glutamatergic synaptic activity, resulting in decreasing the mRNA and protein levels of Arc in SD rats.

The immediate-early gene Arc, which is considered as a plasticity-related gene, plays a key role in the long-term potentiation, long-term depression, and synaptic transmission [46, 47]. Glutamate could increase the mRNA and protein expression of Arc in neurons. The increase of Arc expression in the excitatory neurons was dependent on NMDA receptor, which is one of three types of ionotropic glutamate receptors [48, 49]. In comparison, another ionotropic glutamate receptor, alpha-amino-3-hydroxy-5-methyl-4-isoxazolepropionic acid (AMPA) receptor, was reported to negatively regulate the Arc transcription or not to affect the level of Arc [48, 50]. Given these previous reports, the alteration of Arc expressions in SD and PG-treated SD rats may be mediated via the regulation of NMDA receptor activity.

The NMDA receptor forms a tetramer by three different subunits: NR1, NR2, and NR3. Heterotetrameric NMDA receptor is broadly expressed in the brain, which consisted of two obligatory glycine-binding NR1 subunits and two regionally localized glutamate-binding NR2 subunits, particularly NR2A and NR2B. We examined the effect of PG on the NMDA receptor, focusing on the NR2 subunits containing the glutamate binding site. Previous studies have reported the involvement of the phosphorylation of NR2 subunits in the action of lithium. Lithium inhibited the tyrosine phosphorylation of the NR2A subunit, together with the decreased interaction of NR2A with the Src family tyrosine kinases in the rat hippocampus following cerebral ischemia [51]. Lithium also suppressed the tyrosine phosphorylation of the NR2B subunit in cultured cortical neurons [52]. The tyrosine phosphorylations of NR2A including Y1325 could potentiate the NMDA receptor currents [53, 54]. In comparison, the phosphorylation of NR2B Y1472 inhibited the endocytosis of the NR2B-containing NMDA receptor, leading to the stabilization of the receptor on the cell surface [55]. Interestingly, we found that SD increased the tyrosine phosphorylation of the NR2A subunit (Y1325), whereas PG alleviated the SD-induced phosphorylation. However, the phosphorylation of the NR2B subunit (Y1472) was induced by neither SD nor PG treatment. Therefore, our result indicated that PG might relieve the glutamate neurotransmission activated in SD rats, regulating the potential of the NMDA receptor currents rather than the surface expression or stabilizing of the receptor. There were some limitations in our RNA-seq result. In the DEG analysis, we did not determine the differential expressions by FDR-corrected *p* value <0.05. However, after selecting the candidate genes related to the effect of PG on SD of DEGs, we validated the mRNA expressions of candidate genes using the qRT-PCR as well as the expression levels of proteins encoded by the genes using immunoblotting.

## 5. Conclusions

In the present study, we found that PG could increase the mRNA and protein expressions of Slc1a3 and Slc1a2 in the prefrontal cortex of PG-treated SD rats. The increase of the expressions of Slc1a3 and Slc1a2 by PG may lead to the decrease of the extracellular glutamate levels. In turn, it would reduce the NMDA receptor activation and the expression level of Arc in SD rats. The attenuated glutamatergic neurotransmission by PG would contribute to the reduction of mania-like behaviors in SD rats. These results suggested the useful therapeutic effect of PG on the management of a manic phase of BPD.

## Figures and Tables

**Table 1:** Gene-enrichment analysis on differentially expressed genes (DEGs) by *Panax ginseng* (PG) in sleep deprivation (SD) rats.

KEGG MapID	Map name	No. of genes	Genes	*p* value	FDR *p* value
04724	Glutamatergic synapse	6	Grin3a, Slc1a2, Slc1a3, Grik3, Adcy1, Gng2	< 0.0001	0.0001
04371	Apelin signaling pathway	6	Nos1, Spp1, Prkce, Adcy1, Klf2, Gng2	< 0.0001	0.0002
05418	Fluid shear stress and atherosclerosis	5	Dusp1, Mgst1, Bmp4, Klf2, Bmpr1b	0.0001	0.0027
04062	Chemokine signaling pathway	5	Shc3, Ccl4, Adcy1, Gng2, Gsk3b	0.0001	0.0037
04024	cAMP signaling pathway	5	Grin3a, Pde4d, Camk2a, Atp2b4, Adcy1	0.0001	0.0043
05032	Morphine addiction	4	Pde4d, Gabrb2, Adcy1, Gng2	0.0003	0.0072
04713	Circadian entrainment	4	Nos1, Camk2a, Adcy1, Gng2	0.0003	0.0073
04919	Thyroid hormone signaling pathway	4	Thrb, Bmp4, Pdpk1, Gsk3b	0.0006	0.0105
04722	Neurotrophin signaling pathway	4	Shc3, Camk2a, Pdpk1, Gsk3b	0.0007	0.0110
04550	Signaling pathways regulating pluripotency of stem cells	4	Bmp4, Inhba, Bmpr1b, Gsk3b	0.0009	0.0135
04261	Adrenergic signaling in cardiomyocytes	4	Cacng4, Camk2a, Atp2b4, Adcy1	0.0010	0.0135
04921	Oxytocin signaling pathway	4	Cacng4, Camk2a, Adcy1, Myl6	0.0012	0.0146
04390	Hippo signaling pathway	4	Bmp4, Bmpr1b, Dlg2, Gsk3b	0.0012	0.0146
04360	Axon guidance	4	Camk2a, Bmpr1b, Robo3, Gsk3b	0.0018	0.0192
05200	Pathways in cancer	5	Bmp4, Adcy1, Zbtb16, Gng2, Gsk3b	0.0019	0.0196
04020	Calcium signaling pathway	4	Nos1, Camk2a, Atp2b4, Adcy1	0.0021	0.0199
04510	Focal adhesion	4	Shc3, Spp1, Pdpk1, Gsk3b	0.0026	0.0228
05031	Amphetamine addiction	3	Grin3a, Camk2a, Arc	0.0037	0.0309
04970	Salivary secretion	3	Nos1, Atp2b4, Adcy1	0.0051	0.0402
05206	MicroRNAs in cancer	4	Mir27b, Mir128-1, Mir26a, Prkce	0.0057	0.0419
04925	Aldosterone synthesis and secretion	3	Camk2a, Prkce, Adcy1	0.0058	0.0419
04350	TGF-beta signaling pathway	3	Bmp4, Inhba, Bmpr1b	0.0062	0.0422
04012	ErbB signaling pathway	3	Shc3, Camk2a, Gsk3b	0.0065	0.0422
04727	GABAergic synapse	3	Gabrb2, Adcy1, Gng2	0.0068	0.0422
04080	Neuroactive ligand-receptor interaction	4	Grin3a, Thrb, Gabrb2, Grik3	0.0070	0.0422
04916	Melanogenesis	3	Camk2a, Adcy1, Gsk3b	0.0081	0.0470

Gene-enrichment analysis for DEGs was performed based on the KEGG pathway database (https://www.genome.jp/kegg/). The *p* values were calculated based on a modified Fisher's exact test. The false discovery rate (FDR) was controlled by adjusting the *p* value using the Benjamini-Hochberg algorithm.

**Table 2:** Different expressions of glutamatergic synapse and amphetamine addiction pathway genes in the control, sleep deprivation (SD), and *Panax ginseng* (PG-) treated SD rats.

Gene	Description	FC	
		SD+PG/S	SD/control
Glutamatergic synapse			
Slc1a3	Solute carrier family 1 member 3	5.132	-3.718
Slc1a2	Solute carrier family 1 (glial high-affinity glutamate transporter), member 2	2.181	-1.18
Adcy1	Adenylate cyclase 1 (brain)	3.268	-1.125
Grik3	Glutamate ionotropic receptor kainate type subunit 3	2.032	1.517
Grin3a	Glutamate ionotropic receptor NMDA type subunit 3A	2.012	-1.283
Gng2	G protein subunit gamma 2	62.921	-53.461
Amphetamine addiction			
Camk2a	Calcium/calmodulin-dependent protein kinase II alpha	2.111	-1.135
Arc	Activity-regulated cytoskeleton-associated protein	-2.096	3.674
Grin3a	Glutamate ionotropic receptor NMDA type subunit 3A	2.012	-1.283

FC: fold-change.

## Data Availability

The data used to support the findings of this study are available from the corresponding author upon request.

## Supplementary Materials

Supplementary Materials Supplemental Table S1. Differentially expressed genes between control and sleep deprivation (SD) rats. Supplemental Table S2. Differentially expressed genes between sleep deprivation (SD) and Panax ginseng (PG)-treated SD rats. Supplemental Table S3. Primer sequences used for qRT-PCR.
