# Supplementary material for: Antimania-Like Effect of Panax ginseng Regulating the Glutamatergic Neurotransmission in REM-Sleep Deprivation Rats
Source: Biomed Res Int. 2020 Oct 17;2020:3636874. doi: 10.1155/2020/3636874 (PMC7586145; doi:10.1155/2020/3636874)
Supplement: Supplementary Materials — Supplemental Table S1. Differentially expressed genes between control and sleep deprivation (SD) rats. Supplemental Table S2. Differentially expressed genes between sleep deprivation (SD) and Panax ginseng (PG)-treated SD rats. Supplemental Table S3. Primer sequences used for qRT-PCR. [file 3636874.f1.docx]

Table S1. Differentially expressed genes between control and sleep deprivation (SD) rats

| Transcript ID | Gene Symbol | Description | FC |
| --- | --- | --- | --- |
|  |  |  | SD/control |
| NM_001257349, NM_031754 | Gng2 | G protein subunit gamma 2 | -53.461 |
| NM_001008839 | RT1-CE16 | RT1 class I, locus CE16 | -6.249 |
| NM_001270854, NM_012672 | Thrb | thyroid hormone receptor beta | -5.952 |
| NM_001100983 | Myl6l | myosin, light polypeptide 6, alkali, smooth muscle and non-muscle-like | -4.503 |
| NM_001289941, NM_001289942, NM_001289943, NM_019225 | Slc1a3 | solute carrier family 1 member 3 | -3.718 |
| NM_012681 | Ttr | transthyretin | -3.484 |
| NM_032085 | Col3a1 | collagen type III alpha 1 chain | -3.054 |
| NM_001113223 | LOC100134871 | beta globin minor gene | -2.943 |
| NM_001109486 | Myl6b | myosin, light chain 6B, alkali, smooth muscle and non-muscle | -2.893 |
| NM_001190162, NM_001190163, NM_031511 | Igf2 | insulin-like growth factor 2 | -2.777 |
| NM_177426 | Gstm2 | glutathione S-transferase mu 2 | -2.709 |
| NM_001007738 | Tmem252 | transmembrane protein 252 | -2.677 |
| NM_001008841 | RT1-CE3 | RT1 class I, locus CE3 | -2.665 |
| NM_053933 | Pcdha4 | protocadherin alpha 4 | -2.564 |
| NM_001004095 | S100a11 | S100 calcium binding protein A11 | -2.559 |
| NM_001008847 | RT1-Da | RT1 class II, locus Da | -2.421 |
| NM_198776 | Hbb-b1 | hemoglobin, beta adult major chain | -2.406 |
| NM_031810 | Defb1 | defensin beta 1 | -2.329 |
| NM_053822 | S100a8 | S100 calcium binding protein A8 | -2.267 |
| NM_013122 | Igfbp2 | insulin-like growth factor binding protein 2 | -2.266 |
| NM_001107943 | Acer2 | alkaline ceramidase 2 | -2.24 |
| NM_001100970 | Aebp1 | AE binding protein 1 | -2.18 |
| NM_001077589 | Rgs16 | regulator of G-protein signaling 16 | -2.122 |
| NM_053565 | Socs3 | suppressor of cytokine signaling 3 | -2.11 |
| NM_013015 | Ptgds | prostaglandin D2 synthase | -2.109 |
| NM_001009717 | Lrg1 | leucine-rich alpha-2-glycoprotein 1 | -2.104 |
| NM_001106314 | Ifitm1 | interferon induced transmembrane protein 1 | -2.101 |
| NM_001025002 | LOC310926 | hypothetical protein LOC310926 | -2.09 |
| NM_001105210, NM_001105211 | Mrvi1 | murine retrovirus integration site 1 homolog | -2.08 |
| NM_001012107 | Gpr157 | G protein-coupled receptor 157 | -2.038 |
| NM_017224 | Slc22a6 | solute carrier family 22 member 6 | -2.028 |
| NM_001001972 | Ly6g6e | lymphocyte antigen 6 complex, locus G6E | -2.014 |
| NM_001013101 | Moap1 | modulator of apoptosis 1 | -2.009 |
| NM_053304 | Col1a1 | collagen, type I, alpha 1 | -2.008 |
| NM_001014043 | Sgms2 | sphingomyelin synthase 2 | -2.004 |
| NM_001109484 | Myl6 | myosin light chain 6 | 2.001 |
| NM_001191891 | C1qtnf9 | C1q and tumor necrosis factor related protein 9 | 2.01 |
| NM_001191752 | Ccdc77 | coiled-coil domain containing 77 | 2.011 |
| NM_013165 | Cckbr | cholecystokinin B receptor | 2.016 |
| NR_037356 | Mir3568 | microRNA 3568 | 2.027 |
| NM_001108110 | Ccnt1 | cyclin T1 | 2.092 |
| NR_037395 | Mir3594 | microRNA 3594 | 2.118 |
| NR_031862 | Mir106b | microRNA 106b | 2.121 |
| NM_001256509 | Fosb | FBJ osteosarcoma oncogene B | 2.128 |
| NM_001144861 | Plekha7 | pleckstrin homology domain containing A7 | 2.137 |
| NM_153626 | Npas4 | neuronal PAS domain protein 4 | 2.137 |
| NM_053769 | Dusp1 | dual specificity phosphatase 1 | 2.148 |
| NM_001014271 | LOC367515 | similar to RIKEN cDNA 1700081O22 | 2.18 |
| NR_031873 | Mir128-1 | microRNA 128-1 | 2.187 |
| NM_053930 | Gp1bb | glycoprotein Ib platelet beta subunit | 2.188 |
| NM_001017512 | Zfp61 | zinc finger protein 61 | 2.226 |
| NM_001109127 | Cbln1 | cerebellin 1 precursor | 2.243 |
| NM_001134756, NM_001134757 | Gnpnat1 | glucosamine-phosphate N-acetyltransferase 1 | 2.296 |
| NM_031628 | Nr4a3 | nuclear receptor subfamily 4, group A, member 3 | 2.527 |
| NR_031785 | Mir341 | microRNA 341 | 2.602 |
| NM_024388 | Nr4a1 | nuclear receptor subfamily 4, group A, member 1 | 2.679 |
| NR_032114 | Mir377 | microRNA 377 | 2.765 |
| NM_001106121 | Ucma | upper zone of growth plate and cartilage matrix associated | 2.806 |
| NM_053633 | Egr2 | early growth response 2 | 3.004 |
| NM_022197 | Fos | FBJ osteosarcoma oncogene | 3.103 |
| NM_019328 | Nr4a2 | nuclear receptor subfamily 4, group A, member 2 | 3.121 |
| NM_053942 | Pcdha8 | protocadherin alpha 8 | 3.278 |
| NM_001079885, NM_001198653 | Vwa3a | von Willebrand factor A domain containing 3A | 3.443 |
| NM_001034960 | Spag6 | sperm associated antigen 6 | 3.663 |
| NM_019361 | Arc | activity-regulated cytoskeleton-associated protein | 3.674 |
| NM_001013853 | Hba-a1 | hemoglobin alpha, adult chain 1 | 3.78 |

Listed genes showing fold change (FC) of ≥ 2.0 and the *p*-value of < 0.05 in the comparison between control and SD.

Table S2. Differentially expressed genes between sleep deprivation (SD) and *Panax ginseng* (PG)-treated SD rats

| Transcript ID | Gene Symbol | Description | FC |
| --- | --- | --- | --- |
|  |  |  | SD+PG/SD |
| NM_001170601 | Srp72 | signal recognition particle 72 | -4.011 |
| NM_031688 | Sncg | synuclein, gamma | -3.774 |
| NR_032114 | Mir377 | microRNA 377 | -3.382 |
| NM_001134756, NM_001134757 | Gnpnat1 | glucosamine-phosphate N-acetyltransferase 1 | -2.618 |
| NM_001003403 | Apold1 | apolipoprotein L domain containing 1 | -2.572 |
| NM_001034960 | Spag6 | sperm associated antigen 6 | -2.571 |
| NR_031832 | Mir27b | microRNA 27b | -2.511 |
| NR_032765 | Mir761 | microRNA 761 | -2.414 |
| NM_001037140 | Pcdhga1 | protocadherin gamma subfamily A, 1 | -2.398 |
| NR_037331 | Mir3074 | microRNA 3074 | -2.389 |
| NR_032274 | Mir410 | microRNA 410 | -2.35 |
| NR_037315 | Mir3541 | microRNA 3541 | -2.298 |
| NM_053769 | Dusp1 | dual specificity phosphatase 1 | -2.284 |
| NR_002703 | Rmrp | RNA component of mitochondrial RNA processing endoribonuclease | -2.274 |
| NR_106698 | Mir6326 | microRNA 6326 | -2.265 |
| NR_031873 | Mir128-1 | microRNA 128-1 | -2.228 |
| NM_001109484 | Myl6 | myosin light chain 6 | -2.217 |
| NR_046238 | Rn5-8s | 5.8S ribosomal RNA | -2.204 |
| NM_017259 | Btg2 | BTG family, member 2 | -2.194 |
| NM_001170557 | Plk5 | polo-like kinase 5 | -2.183 |
| NM_001007694 | Ifit3 | interferon-induced protein with tetratricopeptide repeats 3 | -2.18 |
| NM_012812, NR_037674 | Cox6a2 | cytochrome c oxidase subunit 6A2 | -2.177 |
| NM_001106930 | Efcab1 | EF hand calcium binding domain 1 | -2.162 |
| NM_001170482 | Atoh7 | atonal bHLH transcription factor 7 | -2.112 |
| NR_002705 | LOC252890 | Z39 small nucleolar RNA | -2.11 |
| NM_019361 | Arc | activity-regulated cytoskeleton-associated protein | -2.096 |
| NM_001105965 | Dpt | dermatopontin | -2.091 |
| NM_053858 | Ccl4 | C-C motif chemokine ligand 4 | -2.065 |
| NM_001108135 | Robo3 | roundabout guidance receptor 3 | -2.063 |
| NM_033234 | Hbb | hemoglobin subunit beta | -2.063 |
| NR_031947 | Mir421 | microRNA 421 | -2.047 |
| NM_001108279 | Bcl6b | B-cell CLL/lymphoma 6B | -2.036 |
| NM_001007684 | Klf2 | Kruppel-like factor 2 | -2.036 |
| NR_037334 | Mir3556b | microRNA 3556b | -2.031 |
| NR_032772 | Mir496 | microRNA 496 | -2.03 |
| NR_031830 | Mir26a | microRNA 26a | -2.024 |
| NM_147136 | LOC257642 | rRNA promoter binding protein | -2.018 |
| NR_031787 | Mir343 | microRNA 343 | -2.014 |
| NM_214457 | Phactr1 | phosphatase and actin regulator 1 | 2.004 |
| NM_001013101 | Moap1 | modulator of apoptosis 1 | 2.004 |
| NM_053643 | Cds2 | CDP-diacylglycerol synthase 2 | 2.006 |
| NM_053473 | Ppp1r9a | protein phosphatase 1, regulatory subunit 9A | 2.007 |
| NM_001198583, NM_138546 | Grin3a | glutamate ionotropic receptor NMDA type subunit 3A | 2.012 |
| NM_134349 | Mgst1 | microsomal glutathione S-transferase 1 | 2.014 |
| NM_022282 | Dlg2 | discs large MAGUK scaffold protein 2 | 2.021 |
| NM_023975 | Wdr7 | WD repeat domain 7 | 2.022 |
| NM_001112716, NM_181373 | Grik3 | glutamate ionotropic receptor kainate type subunit 3 | 2.032 |
| NM_024361 | Ndst1 | N-deacetylase/N-sulfotransferase 1 | 2.032 |
| NM_080692 | Cacng4 | calcium voltage-gated channel auxiliary subunit gamma 4 | 2.045 |
| NM_017171 | Prkce | protein kinase C, epsilon | 2.049 |
| NM_012778 | Aqp1 | aquaporin 1 | 2.049 |
| NM_133536 | Rab3c | RAB3C, member RAS oncogene family | 2.052 |
| NM_012957 | Gabrb2 | gamma-aminobutyric acid type A receptor beta 2 subunit | 2.053 |
| NM_053933 | Pcdha4 | protocadherin alpha 4 | 2.054 |
| NM_001014099 | Paqr8 | progestin and adipoQ receptor family member 8 | 2.056 |
| NR_106672 | Snora58 | small nucleolar RNA, H/ACA box 58 | 2.059 |
| NM_017323 | Nr2c2 | nuclear receptor subfamily 2, group C, member 2 | 2.06 |
| NM_013066 | Map2 | microtubule-associated protein 2 | 2.067 |
| NM_032080 | Gsk3b | glycogen synthase kinase 3 beta | 2.068 |
| NM_001191943 | Dok6 | docking protein 6 | 2.07 |
| NR_037394 | Mir3593 | microRNA 3593 | 2.08 |
| NM_001113328, NM_001113329, NM_001113332, NM_001113334, NM_017032 | Pde4d | phosphodiesterase 4D, cAMP-specific | 2.084 |
| NM_012920 | Camk2a | calcium/calmodulin-dependent protein kinase II alpha | 2.111 |
| NM_145765 | Tnfsf15 | tumor necrosis factor superfamily member 15 | 2.112 |
| NM_001013181 | Zbtb16 | zinc finger and BTB domain containing 16 | 2.117 |
| NM_001077201 | Caln1 | calneuron 1 | 2.134 |
| NM_017128 | Inhba | inhibin beta A subunit | 2.144 |
| NM_001107671 | Plcxd3 | phosphatidylinositol-specific phospholipase C, X domain containing 3 | 2.155 |
| NM_001071777 | Gpr17 | G protein-coupled receptor 17 | 2.162 |
| NM_001035233, NM_001302089, NM_017215 | Slc1a2 | solute carrier family 1 (glial high affinity glutamate transporter), member 2 | 2.181 |
| NM_052799 | Nos1 | nitric oxide synthase 1 | 2.182 |
| NM_001170799 | Ipcef1 | interaction protein for cytohesin exchange factors 1 | 2.186 |
| NM_022178 | Myo5a | myosin VA | 2.219 |
| NM_207616 | Hyal1 | hyaluronoglucosaminidase 1 | 2.223 |
| NM_017357 | Hpcal4 | hippocalcin-like 4 | 2.223 |
| NM_001024259 | Bmpr1b | bone morphogenetic protein receptor type 1B | 2.252 |
| NM_031081 | Pdpk1 | 3-phosphoinositide dependent protein kinase-1 | 2.272 |
| NM_053504 | Prss12 | protease, serine 12 | 2.353 |
| NM_001005871 | Atp2b4 | ATPase plasma membrane Ca2+ transporting 4 | 2.366 |
| NM_199504 | Pcdha2 | protocadherin alpha 2 | 2.388 |
| NM_001105743 | Shc3 | SHC adaptor protein 3 | 2.405 |
| NM_001109147 | Klf13 | Kruppel-like factor 13 | 2.414 |
| NR_106707 | Mir6333 | microRNA 6333 | 2.573 |
| NM_001142652 | Neurl1b | neuralized E3 ubiquitin protein ligase 1B | 2.6 |
| NM_001109486 | Myl6b | myosin, light chain 6B, alkali, smooth muscle and non-muscle | 2.629 |
| NM_001170334 | Gfod1 | glucose-fructose oxidoreductase domain containing 1 | 2.643 |
| NM_012881 | Spp1 | secreted phosphoprotein 1 | 2.671 |
| NM_001135600 | Cyp4v3 | cytochrome P450, family 4, subfamily v, polypeptide 3 | 2.769 |
| NM_012827 | Bmp4 | bone morphogenetic protein 4 | 2.779 |
| NM_001107239 | Adcy1 | adenylate cyclase 1 (brain) | 3.268 |
| NM_001289941, NM_001289942, NM_001289943, NM_019225 | Slc1a3 | solute carrier family 1 member 3 | 5.132 |
| NM_001100983 | Myl6l | myosin, light polypeptide 6, alkali, smooth muscle and non-muscle-like | 9.196 |
| NM_001270854, NM_012672 | Thrb | thyroid hormone receptor beta | 9.777 |
| NM_001257349, NM_031754 | Gng2 | G protein subunit gamma 2 | 62.921 |

Listed genes showing fold-change (FC) of ≥ 2.0 and the *p*-value of < 0.05 in the comparison between SD and PG-treated SD.

Table S3. Primer sequences used for qRT-PCR

| Gene | Forward (5’ to 3’) | Reverse (5’ to 3’) |
| --- | --- | --- |
| Slc1a3 | GGACACCATTATGCTAAC | ATGTAACGCTCAGAATCT |
| Slc1a2 | TTATGACGACACGAAGAA | CATCTATTACGACAGAGTTG |
| Adcy1 | ATCTCTATTACCAGTCCTA | CTATGTAGAAGTCGTTGA |
| Grik3 | TTCTGCCAATATCATCAAC | GAAGTGAATCCTCTGAATG |
| Grin3a | CCTATGACTAACGACTCTT | ACTTCTTGAACTTGATTGG |
| Gng2 | AAGAAGAAGGACAAGTATC | GAGACACAATAGACACATA |
| Camk2 | GCACCACTACCTTATCTT | AGCCTCACTGTAATACTC |
| Arc | GCTGACACCTACAATATCA | GTTATCCACTCTGCTCTC |
| Ubc | AGAAGGTCAAACAGGAAGATA | CCAAGAACAAGCACAAGAA |
